# Recent Progress of Flower Colour Modification by Biotechnology

**DOI:** 10.3390/ijms10125350

**Published:** 2009-12-15

**Authors:** Yoshikazu Tanaka, Filippa Brugliera, Steve Chandler

**Affiliations:** 1 Institute for Plant Science, Suntory Holdings Ltd., 1-1-1 Wakayamadai, Shimamoto, Mishima, Osaka 618-8503, Japan; 2 Florigene Pty Ltd., 1 Park Drive, Bundoora, Victoria 3083, Australia; E-Mails: fbrugliera@florigene.com.au (F.B.); schandler@florigene.com.au (S.C.)

**Keywords:** anthocyanin, flavonoid, flower colour, genetic engineering, genetically modified organism (GMO)

## Abstract

Genetically-modified, colour-altered varieties of the important cut-flower crop carnation have now been commercially available for nearly ten years. In this review we describe the manipulation of the anthocyanin biosynthesis pathway that has lead to the development of these varieties and how similar manipulations have been successfully applied to both pot plants and another cut-flower species, the rose. From this experience it is clear that down- and up-regulation of the flavonoid and anthocyanin pathway is both possible and predictable. The major commercial benefit of the application of this technology has so far been the development of novel flower colours through the development of transgenic varieties that produce, uniquely for the target species, anthocyanins derived from delphinidin. These anthocyanins are ubiquitous in nature, and occur in both ornamental plants and common food plants. Through the extensive regulatory approval processes that must occur for the commercialization of genetically modified organisms, we have accumulated considerable experimental and trial data to show the accumulation of delphinidin based anthocyanins in the transgenic plants poses no environmental or health risk.

## 1. Introduction

Flavonoids, carotenoids and betalains are major floral pigments [1]. Between these three groups, the flavonoids contribute most to the range and type of coloured pigments in plants. As genetic engineering techniques have primarily targeted the flavonoid pathway as a means to alter flower colour, we will concentrate only on flavonoid pathway modification in this review. Flavonoids consist of more than 10 classes of compounds. Anthocyanins confer orange, red, magenta, violet and blue colours. Aurones and chalcones are yellow pigments while flavones and flavonols are colourless or very pale yellow, at least to the human eye.

The flavonoid biosynthetic pathway leading to floral pigment accumulation have been well characterized and the genes encoding relevant enzymes and transcriptional factors have been isolated [1,2]. Many studies of flower colour modification by genetic engineering have been performed as outlined in a recent review [3]. In this review, we will describe recent progress towards a further understanding of the contribution of flavonoid biosynthesis and cell physiology in the regulation of flower colour and discuss some examples of flower colour modification in important flower species.

## 2. Recent Progress; Anthocyanin Biosynthesis and Flower Colour

### 2.1. Anthocyanin Structure and Colour

The final flower colour in those species in which colour is primarily derived from anthocyanins is determined by a combination of various factors. As well as anthocyanin structure, type and concentration, co-existing compounds (co-pigments), metal ion type and concentration, pH of vacuoles, anthocyanin localization and shapes of surface cells all contribute to final flower colour [4]. Through combinations of these factors, plant species have evolved flower colours to attract insect pollinators.

**Scheme 1.**
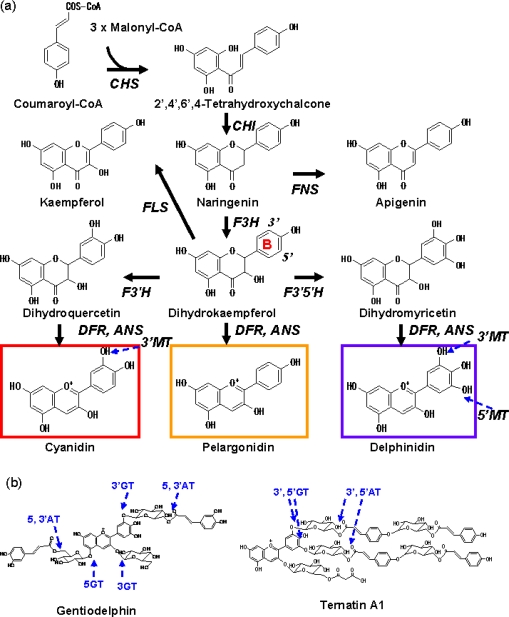
(a) The biosynthetic pathway leading to the biosynthesis of the anthocyanidin. Anthocyanidin is further modified with glycosyl, acyl, or methyl groups catalysed by glycosyltransferase, acyltransferase and methyltransferase as described in the text. The typical color that each anthocyanidin tend to give is shown, but the actual color depends on various factors as described in the text. Note that though the positions of methylation on the B-ring are shown, methyl groups are added to anthocyanins, not to anthocyanidins. Abbreviations: CHS, chalcone synthase; F3H, flavanone 3-hydroxylase; F3′H, flavonoid 3′-hydroxylase; F3′5′H, flavonoid 3′,5′-hydroxylase; DFR, dihydroflavonol 4-reductase; ANS, anthocyanidin synthase; MT, methyltransferase, GT, glucosyltransferase; AT, acyltransferase; FNS, flavone synthase; FLS, flavonol synthase. (b) Typical blue polyacyl anthocyanins; gentiodelphin from *Gentiana triflora* and ternatin A1 from *Clitoria ternatea*.

Though hundreds of anthocyanins have been reported [5], they are primarily based upon six common anthocyanidins (chromophores of anthocyanins); pelargonidin, cyanidin, peonidin, delphinidin, petunidin and malvidin. In terms of biosynthesis, since peonidin is derived from cyanidin and petunidin and malvidin are both derived from delphinidin, there are only three major anthocyanidins; pelargonidin, cyanidin and delphinidin (Scheme 1). Blue flowers tend to have delphinidin and its derivatives and intense red flowers tend to have pelargonidin as the anthocyanidin base. An increase in the number of hydroxyl groups on the B-ring (Scheme 1) imparts a bluer colour to the anthocyanins derived from the anthocyanidin, while methylation of the 3’ or 5’-hydroxyl group (Scheme 1) results in a slight reddening.

### 2.2. Anthocyanin Modification

Anthocyanidins are modified with glycosyl or acyl moieties in a species specific manner by specific glycosyltransferases and acyltransferases. Aromatic acylation of anthocyanins shifts their colour toward blue and increases their stability.

Anthocyanins containing plural aromatic acyl groups are called polyacylated anthocyanins and exhibit blue colour as a result of intra-molecular stacking [4,6]. A well studied example is a gentian anthocyanin, gentiodelphin, in which the caffeoyl moiety at the 3’ position of gentiodelphin has been shown to contribute to intra-molecular stacking, resulting in the stable blue colour of the flowers [7]. In terms of biosynthesis, acyl groups are transferred to glycosyl moieties of anthocyanins and thus glycosylation precedes acylation.

#### 2.2.1. Glycosylation of Anthocyanins

The anthocyanin biosynthetic pathway leading to anthocyanidin 3-glucosides is well conserved and many plant species produce anthocyanidin 3,5-diglucosides and their respective derivatives. In general, 3-glucosylation occurs prior to 5-glucosylation and the two glucosylations are separately catalyzed, by UDP-glucosyl dependent anthocyanidin 3-glucosyltransferase (3GT) and anthocyanin 5-glucosyltransferase (5GT), respectively. Many 3GT and 5GT genes have been isolated and they form two distinct clusters within the family 1 glycosyltransferase [1]. Rose petals predominantly contain cyanidin or pelargonidin 3,5-diglucoside and a relatively small amount of cyanidin or pelargonidin 3-glucoside. Ogata *et al.* reported that *Rosa hybrida* petals have a unique UDP-glucose dependent anthocyanidin 5,3-glucosyltransferase and have isolated the corresponding cDNA clone [8]. Unusually, the enzyme catalyzes 5-glucosylation of anthocyanidins followed by 3-glucosylation of anthocyanidin 5-glucoside. In addition rose petals also contain an anthocyanidin 3-glucosyltransferase belonging to the 3GT cluster of genes [9].

Interestingly, butterfly pea (*Clitoria ternatea*) contains an anthocyanin 3′,5′-glucosyltransferase (3′,5′-GT [10], Scheme 1b) gene whose amino acid sequence is closely related to 3GT [11]. This indicates the 3′,5′-GT gene possibly evolved from the 3GT gene in butterfly pea. On the other hand the gentian anthocyanin 3′-glucosyltranferase (3′GT) belongs to a different GT clade (3′,7GT clade) from anthocyanidin 3GT [12] suggesting anthocyanin 3′-GT activity independently acquired in butterfly pea and gentian.

#### 2.2.2. Acylation of Anthocyanins

The gene encoding an enzyme that catalyzes the transfer of aromatic acyl groups to anthocyanins was first isolated from gentian, *i.e.*, hydroxycinnamoyl CoA: anthocyanin 5-glucoside hydroxycinnamoyl CoA transferase (5AT) [13]. Extensive biochemical analysis *in vitro* revealed that the same enzyme catalyzes aromatic acylation of glucosyl moieties at both the 5 and 3’ positions [14]. A hydroxycinnamoyl CoA: anthocyanin 3-glucoside hydroxycinnamoyl CoA transferase gene has now been isolated from perilla (*Perilla frutescens*) [15]. Several aliphatic acyltransferase genes have been also isolated as summarized [16]. All belong to the BAHD (benzylalcohol *O*-acetyltransferase (BEAT), anthocyanin *O*-hydroxycinnamoyltransferase (AHCT), anthranilate *N*-hydroxycinnamoyl/benzoyltransferase (HCBT) and deacetylvindoline 4-*O*-acetyltransferase (DAT)) type acyl CoA dependent acyltransferase group [17].

Recently, a number of acylglucose dependent anthocyanin acyltransferase genes have been isolated as shown below. All have serine carboxypeptidase-like sequences. *Arabidopsis* sinapoylglucose acyltransferase is required for the synthesis of the sinapoylated anthocyanins such as cyanidin 3-(6-(4-glucosyl-*p*-coumaroyl)-2-(2-sinapoyl-xylosyl)glucosyl)-5-(6-malonyl-glucoside) [18] while transfer of *p*-coumaroyl and malonyl moieties is catalyzed by BAHD type acyl CoA dependent [16]. Butterfly pea produces blue polyacylated anthocyanins such as ternatin A1 (Scheme 1b) and its derivatives and it has been shown that losing acyl groups resulted in mauve colour rather than blue [6,19]. Noda *et al*. purified a butterfly pea aromatic acyltransferase that catalyzed the transfer of an aromatic acyl group from acylglucose to both the 3’ and 5’-glucosides of anthocyanins and isolated the corresponding cDNA clone [20]. Carnation petals generally accumulate macrocyclic anthocyanins 3,5-di-*O*-glucoside-(6″,6‴-malyl diester) of cyanidin or pelargonidin. A malylglucose dependent anthocyanin malyltransferase has been partially purified from carnation petals that have malylated anthocyanins [21] and the corresponding cDNA clone has been also isolated [22]. These studies indicate that plants have acquired enzymes exhibiting suitable substrate specificity through gene duplication and neo-functionalization of various pre-existing genes.

#### 2.2.3. Methylation of Anthocyanins

Anthocyanin methylation adds structural and colour diversity to anthocyanins. The cDNA clones corresponding to *S*-adenosyl methionine dependent anthocyanin methyltransferase (MT) have been isolated from petunia and torenia flowers [23] and from grape berries [24]. Interestingly these genes belong to the cation dependent type II MT group, whereas flavone or flavonol MTs usually belong to type I MT.

### 2.3. Metal Ions

The role of metal ions in blue flower colours have been clarified in recent years and has been summarized in reviews [4,25]. Shiono *et al.* determined the X-ray chrystallographic structure of protocyanin (*Centaurea cyanus* (corn flower) anthocyanin flavone complex) to show that protocyanin consists of six molecule of cyanidin 3-(6-succinylglucoside)-5-glucoside, six molecules of apigenin 7-glucuronide-4′-(6-malonylglucoside), one Fe^3+^, one Mg^2+^ and two Ca^2+^ ions. The X-ray crystallographic structure of commelinin from *Commelina communis* (Asiatic dayflower) has been also determined. *In vitro* reconstituted commelinin consists of six molecules of delphinidin 3-(6-*p*-coumaroylglucoside)-5-(6-malonylglucoside), six molecules of flavone glucoside (6-*C*-glucosylgenkwanin 4’-glucoside), and two Mg^2+^ ions [26], while native commelinin consists of six molecules of anthocyanins, six molecules of flavone glucoside, and four Mg^2+^ ions [27]. The two newly identified Mg atoms are proposed to stabilize the superstructure of commelinin.

Extensive chemical studies of hydrangea petal-like sepals revealed the presence of delphinidin-based anthocyanins, 5-*O*-acylquinic acid and Al^3+^ ions. A higher vacuolar pH (around 4.0 rather than 3.0) is necessary for the blue flower colours [4,28]. The lower sections of the petals of some tulip cultivars are blue while the remainder of the petal is purple. Shoji *et al.* revealed that the cells in the blue region contain about 9.5 mM of Fe^3+^ (25 times higher than purple coloured cells) while there are no significant differences in anthocyanin type (delphinidin 3-rutinoside) or concentration, flavonol type (quercetin glucosides) or concentration or vacuolar pH. The amount of Fe^3+^ ions therefore appears to play a critical role in development of the blue colour [29]. Further investigation has recently revealed that a homologue of *Arabidopsis VIT1* (vacuolar iron transporter) is highly expressed in the blue part of the tulip petals [30].

### 2.4. Regulation of Vacuolar pH

Anthocyanins are a red colour under low pH environments and a blue colour under neutral or alkaline pH environments. Since anthocyanins localize in vacuole, vacuolar pH is important for anthocyanin colour. Vacuolar pH is generally regulated by vacuolar ATPase and pyrophosphase in plant cells. Additionally morning glory and petunia have been recently shown to have unique pumps to carry H^+^ to or from the vacuoles as shown below.

The petal colour of the Japanese morning glory (*Ipomoea nil*) changes from purple to blue during flower opening. Mutants, in which this colour change does not occur, are called *purple*. The vacuolar pH of purple and blue cells of petals *I. tricolor* were shown to be pH 6.6 and 7.7, respectively, while there was no changes in anthocyanin type or concentration in these cells [31]. *Purple* was shown to encode a protein that was homologous to a sodium proton antiporter and was highly expressed before flower opening [32]. The antiporter was later shown to exchange K^+^ with H^+^ rather than Na^+^ with H^+^ and this exchange contributes to flower colour change to blue and petal opening [33].

In petunia, mutation of any of the *PH1* to *PH7* loci results in flowers with a blue color rather than a purple colour and increased petal pH homogenates. This is the result of inhibition of vacuolar acidification. *PH4* encodes a *Myb* type transcription factor and the role of this factor in the regulation of vacuolar pH and anthocyanin biosynthesis has been discussed in a recent review [34]. *PH5* has been shown to encode a P_3A_-H^+^-ATPase localized on the vacuolar membrane while other members of the P_3A_-H^+^-ATPase family are believed to be plasma-membrane-localized proton pumps [35].

## 3. B-Ring Hydroxylation

### 3.1. F3′H and F3′5′H; Key Players of Flower Colour Modification

**Figure 1.**
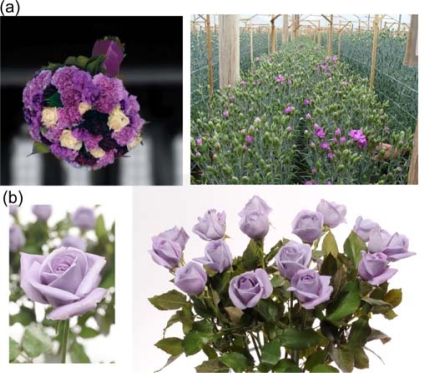
Commercialized transgenic carnation (a) and rose (b) cultivars producing delphinidin and having blue hue that hybridization breeding has not achieved. The transgenic carnation and rose also contain selectable marker genes; for herbicide resistance in carnation and antibiotic resistance in rose.

As described above, more and more potentially useful molecular tools to engineer blue or red flower colours are becoming available. However, only modification of B-ring hydroxylation has been practically demonstrated in changing flower colour. B-ring hydroxylation of flavonoids is catalyzed by the two cytochrome P450 type mono-oxygenases, flavonoid 3′-hdroxylase (F3′H) and flavonoid 3′,5′-hydroxylase (F3′5′H). (Figure 1). In cytochrome P450 nomenclature (http://drnelson.utmem.edu/CytochromeP450.html), they are designated as CYP75B and CYP75A, respectively. The genes encoding these two enzymes were first isolated from petunia, followed by many plant species. Phylogenetic analysis suggests that the genes encoding F3′H and F3′5′H diverted before speciation of flowering plants [36]. However, some chrysanthemum family species (aster, osteospermum, cineraria) which produce delphinidin based anthocyanins contain a CYP75B type F3′5′H and not the CYP75A type, indicating the chrysanthemum family once lost the F3′5′H gene during their evolution. F3′5′H function was reacquired by gene duplication and neo-functionalization of the CYP75B-type gene [37]. It has been also shown that only a few amino acid substitutions in the C-terminal region could functionally change F3′H to F3′5′H [38] illustrating the relatively high plasticity of the flavonoid biosynthetic pathway.

### 3.2. Natural Mutations of F3′H and F3′5′H

The effect of B-ring hydroxylation on flower colour was clearly shown in mutants of these genes, as described below. The locus *magenta* (*mg*) in *I. nil* results in production of magenta flowers containing pelargonidin derivatives while the wild-type produces blue flowers containing peonidin derivatives, indicating that *Mg* regulates flavonoid 3′-hydroxylation. Molecular analysis of *mg* revealed that nucleotide substitution generated a stop codon, disrupting F3′H function. In *I. purplea*, a similar colour change was caused by a transposon insertion in the F3′H gene [39]. Species of *Ipomoea* from the *Mina* section, such as *I. quamoclit*, exhibit red flowers producing pelargonidin derived anthocyanins, which are pollinated by hummingbirds. However, blue flowered *Ipomoea* species are pollinated by bees. F3′H gene expression is dramatically reduced in *I. quamoclit* (however, the coding sequence has a F3′H catalytic activity *in vitro*) and its DFR has lost the ability to catalyze dihydroquercetin, a precursor of cyanidin [40].

Blue gentian accumulates delphinidin derivatives, whilst pink gentian (*Gentiana scabra*) cultivars which were bred from two independent mutants accumulate cyanidin based anthocyanins. In the pink varieties, *F3’5’H* transcripts were interrupted due to the insertion of transposable elements into the F3′5′H gene [41].

## 4. Flower Colour Modification Using Genetic Engineering

### 4.1. Lessons from Model Plant Experiments

Petunia, tobacco and torenia have been model species for the study of flower colour modification via genetic engineering, largely because they are easy to transform. Petunia does not accumulate pelargonidin based anthocyanins because its DFR does not catalyze dihydrokaempferol [42] (Scheme 1a). The first successful experiment aimed at manipulation of anthocyanin metabolism in plants was the production of pelargonidin based anthocyanins achieved by expression of a maize *DFR* in a petunia variety that was deficient in F3′5′H and F3′H activities [43].

Since this first experiment many kinds of transgenic plants with altered flower colours have been reported. Transgenic plants with white flowers have been obtained for many plant species by down regulating anthocyanin biosynthetic genes, as summarized in a recent review [3]. In torenia, transcription of double strand RNA (RNAi) has been shown to be a more effective method to down-regulate a target gene than antisense or sense suppression [44]. In this case however, the white colour was not always stable. This was especially notable when the plants were grown in an outdoor field trial in Australian summer conditions (unpublished results of Florigene Pty. Ltd.). In some cases therefore, alternative methods to knock out the function of a gene is more desirable. Direct genome modification utilizing zinc finger nucleases [45,46] may pave the way to achieve irreversible gene disruption, and so stably altered phenotypes.

In order to redirect flavonoid biosynthesis to an extent necessary to achieve a desirable flower colour change, it is not only the over-expression of the gene of a key enzyme on a new pathway that is necessary but also selection of a host that has the proper genetic background. This selection is to minimize competition of endogenous pathways with the introduced enzyme or to allow down-regulation a competing pathway. It is also important to choose a host cultivar with good commercial characteristics. In the next sections we will refer to actual examples of colour modification of flower colour, focusing on the commercially important cut flowers, carnation and rose.

### 4.2. Develpoment of the Moon® Series of Carnation

Over-expression of a petunia F3′5′H gene under the control of a constitutive promoter in a pelargonidin producing carnation variety produces petals in which delphinidin derivatives contribute to about 70% of total anthocyanins [47]. However, there was only a slight colour change toward blue. In order to increase the content of delphinidin derived anthocyanins it was necessary to avoid competition for substrates between two key endogenous enzymes of the anthocyanin pathway (DFR and F3′H) and the introduced enzyme (F3′5′H). To achieve this white carnation cultivars were selected that were specifically deficient in the DFR gene [48]. Expression of petunia F3′5′H (under the control of a promoter region from the snapdragon CHS gene) and petunia DFR (under the control of a constitutive promoter) genes in one such DFR mutant resulted in exclusive accumulation of delphinidin derivatives and significant colour change toward blue (FLORIGENE®Moondust™, Figure 1; the first genetically modified floricultural crop to be sold in the world). Expression of a pansy F3′5′H gene (under the control of a promoter region from the snapdragon CHS gene) and a petunia *DFR-A* gene (under the control of its own promoter and terminator regions) resulted in transgenic plants which also exclusively accumulated delphinidin but at a higher concentration. These flowers had a dark violet colour (FLORIGENE®Moonshadow™, Figure 1).

The same gene combinations were subsequently introduced into a white standard-type carnation (also a mutant in DFR). In these transgenic plants different amounts of delphinidin based pigments accumulated in the petals, depending on the transgenic events. Four events that exhibited stable colour were selected and have now been sold for more than eight years FLORIGENE®Moonvista™, FLORIGENE®Moonshade™, FLORIGENE®Moonlite™ and FLORIGENE®Moonaqua™ (Figure 1, [49]).

Petunia has a cytochrome *b_5_* that specifically transfers electrons to F3′5′H by which petunia can efficiently synthesize 3′,5′-hydroxylated flavonoids [50]. Expression of a petunia F3′5′H (*Hf1*) gene along with a petunia cytochrome *b_5_* gene in a carnation cultivar producing cyanidin derivatives resulted in efficient production of delphinidin based anthocyanins and subsequent colour change petals [51,52]. In this case, it was not necessary to use a carnation line mutant in DFR to achieve exclusive accumulation of delphinidin. The result indicated that enhancement of electron transfer system to F3′5′H sufficiently out competed the endogenous F3′H (and DFR) activities.

### 4.3. Regulatory Issues and Production of the Moon® Carnation

The transgenic carnations described above are grown in Ecuador, Colombia and Australia, and there is now a ten year history of safe use. Production is now consolidated in South America, from where there are good air-freight distribution systems to Europe, Japan and North America. In the course of obtaining the various regulatory approvals necessary for global trading in the transgenic carnation, it has become accepted by regulators that accumulation of delphinidin-based anthocyanins in transgenic carnation plants poses no environmental or health risk [53–55]. In this section we briefly describe the basis for this conclusion.

#### 4.3.1. The Biology of the Parent Organism

Carnations are double-flowered cultivars of hybrids involving two or more *Dianthus* species, one of which is likely to be *Dianthus caryophyllus* [56–58]. The centre of biodiversity for *Dianthus* is southern Europe and the greatest range of *Dianthus* species are found in the south eastern European countries. *D. caryophyllus* is only found wild in coastal Mediterranean areas. Despite decades of cultivation, and plantings in parks and gardens, carnation has not become a weed, or escaped from cultivation, anywhere in the world. We have carried out detailed surveys in and around flower production areas in Colombia, including the location at which the transgenic carnation is currently grown and composted. No carnation plants have been found outside of cultivation [59]. The cultivated carnation has no capacity to escape from cultivation as the crop possesses no vegetative propagation mechanisms and there are no opportunities for seed-set.

Carnation is not a toxic plant and there are very limited reports of allergenic effects relating to the handling of carnation flowers [60,61]. As carnation is insect pollinated in the wild and does not produce air-borne pollen, respiratory allergenicity has not been reported.

#### 4.3.2. The Modified Phenotype

Delphinidin based anthocyanins are ubiquitous in both ornamental plants and common food plants. Homologues of the anthocyanin biosynthesis genes in transgenic carnation are found in all plants producing delphinidin-related anthocyanins, including raw foods such as blueberries, black currants, grapes, eggplants, red currants, cherries and cranberries.

Dietary surveys estimate that the daily consumption of delphinidin is ~2.5–25 mg per person [62] and higher in some individual diets because of the high concentrations of certain foods. For example, in Finland the estimated intake is approximately 80 mg per day [63]. The levels of delphinidin in the transgenic carnation flowers are within the range of these and other common, widely cultivated plants [54,64,65].

#### 4.3.3. Potential for Gene Flow from Transgenic Carnation to Native Plants

An assessment of the potential for gene flow is central to the risk assessment associated with the release of a genetically modified plant [66,67]. In the case of cultivated carnation, there are no realistic avenues for gene flow to occur.

The dispersal of genes from either cultivated plants or imported cut flowers of carnation could theoretically be via vegetative spread leading to the formation of wild populations, formation of seed by a recipient plant (fertilized by pollen dispersed from the transgenic plants) or the formation and dispersal of seed on the transgenic plants. The possibility of any of these events occurring is negligible.

1. Vegetative spread. Carnation does not spread vegetatively as it does not produce organs such as stolons, root-borne shoots, tubers, bulbs or runners. Cuttings must be struck in optimized conditions and roots do not form on discarded tissues. According to the numerous floras we have reviewed, including refuse sites [68] cultivated carnation has never been found growing wild, and efforts to locate wild carnation in production areas have been unsuccessful [59]. In experiments carried out in Colombia we have found that carnation deliberately planted in uncultivated habitats is unable to survive in competition with other plants.
2. Formation of seed by a recipient plant, fertilized by transgenic pollen. Introgression of genes from cultivated crops to wild relatives is well known [69] and in the case of transgenic carnation, which carries a herbicide resistance gene, it has been necessary to evaluate the potential of introgression to wild *Dianthus* populations. This evaluation has concluded that there is no realistic potential for introgression because;
  - In nature, *Dianthus* is insect-pollinated, and pollen is not spread by wind. Hybridization of *Dianthus* is only carried out by the *Lepidoptera* (butterflies, moths) which have proboscis long enough (up to 2.5 cm) to reach the nectaries, which are located right at the base of the flower in all *Dianthus* species [70]. Cultivated carnations are double-flowered and the many petals present on the flowers make it impossible for insects to access any pollen. No hybrid between carnation and any other *Dianthus* species has ever been recorded in the wild.
  - Some cultivars of the cultivated carnation produce little or no pollen [71,72].
  - Assuming, theoretically, an insect were to access a carnation flower in production, or in a vase in someone’s home, the probability of subsequently fertilizing a recipient, flowering, *Dianthus* plant is limited. The *Dianthus* genus is not native to the production areas in South America, and though *Dianthus* species related to carnation are occasionally found they are rare—no *Dianthus* species were found in surveys of flower growing areas [59].
3. Formation and dispersal of seed. The process of seed development takes at least 5 weeks in carnation [73,74] and as cut flowers only survive 3 to 4 weeks at most in the hands of the consumer, seed development on a cut flower imported for consumption is not possible. In the production areas, the same argument can be raised, because the purpose of production is of course flower harvest, which in its own right eliminates the possibility of seed formation in the crop.

#### 4.3.4. Comparative Trials

Comparative trials between the transgenic lines and the parent variety used for transformation are important, in order to ensure there have been no unexpected effects of the transformation process, induced by somaclonal variation. In the case of the transgenic carnation such trials have been carried out in Japan, Australia, Colombia and Europe. The purpose of the trials has been to select lines in which there have been no significant morphological changes in comparison to the controls, particularly in characters which would have a negative impact on the commercial quality of the flower. In carnation, the primary characteristics determining quality are stem length, vase life, number of petals per flower, flower size and, for spray carnations, number of flower buds per stem.

### 4.4. Transgenic Rose

#### 4.4.1. Delphinidin Production and Flower Colour Modification

Roses are the most important cut flower commercially and have played a major role in human culture and artistry from ancient times. Wild roses usually have pink (these flowers contain anthocyanins derived from cyanidin) or white flowers. The cultivated roses we know today (*Rosa hybrida*) are the product of extensive inter-specific hybridization utilising wild species including yellow-flowered (producing carotenoids) and orange-flowered (producing pelargonidin related anthocyanins) species. However, despite the huge range of flower colours that have been bred, rose lack any varieties in the bluish range of flower colour because the genus *Rosa* does not have the biochemical pathway leading to delphinidin-based anthocyanins.

Expression of a F3′5′H gene from petunia, gentian or butterfly pea in rose resulted in no or little delphinidin accumulation in the petals of transgenic plants, even though these genes were shown to be functional in petunia, carnation or yeast. In contrast, expression of pansy (*Viola spp*) F3′5′H genes in rose resulted in a significant amount of delphinidin derived anthocyanins accumulating in petals of the transgenic plants [75]. We do not know the reason why only the pansy F3’5’H genes worked well in rose.

Rose cultivars that have higher vacuolar pH, large amount of flavonols (co-pigments) and weak or no F3’H activity were selected in order to enhance the blue hue of the transgenic petals and to achieve high content of delphinidin. Expression of pansy F3′5′H genes in such cultivars resulted in transgenic lines in which 95% of the anthocyanidins was delphinidin. The colour of the flowers in these lines were of a significantly bluer hue than any conventionally bred cultivar [76]. In recent experiments we have expressed a torenia anthocyanin 5AT gene in addition to the pansy F3′5′H. However, only a fraction of anthocyanins were modified and the acylation did not significantly affect the flower colour. Nevertheless, two selected lines from these experiments (WKS82-130-4-1 and WKS82-130-9-1) were selected for commercial release and processed through the regulatory procedures that we will refer to later (Section 4.4.2).

In order to out compete the endogenous rose DFR for substrates it was targeted for down regulation via RNAi mediated silencing .The resulting transgenic plants, in which pansy F3′5′H and Dutch iris DFR genes were also expressed, produced flowers that accumulated delphinidin-based anthocyanins exclusively with a concomitant colour change towards blue. In breeding experiments, pollen donated from a transgenic line was used in hybridization with a deep red, cyanidin producing rose cultivar. Progeny carrying the transgenes also exclusively contained delphinidin-derived anthocyanins however the amount of delphinidin varied amongst the progeny [76]. The flower colours obtained amongst the progeny was mostly magenta hues, which was primarily attributed to the lower vacuolar pH of the parent. Though, the results show that the complete conversion of the pathway (*i.e.*, exclusive accumulation of delphinidin-related anthocyanins) is heritable irrespective of genetic background, this specific modification of the pathway also conferred growth retardation and so was not suitable for commercialization.

As we mentioned above, blue flower colour is usually the additive result of various factors, such as anthocyanin modification, co-pigment concentration, vacuolar pH and metal ion type and concentration. Targeting these factors in addition to delphinidin production in transgenic carnation and rose is expected to yield a bluer flower colour.

#### 4.4.2. Regulatory Procedures of Transgenic Rose

Regulatory procedures and practice vary to a large degree between countries and territories. In Japan, the transgenic rose is not considered a food or feed and so is largely examined in the context of possible effects on biodiversity in Japan, consistent with the application of the principles of under the Cartagena Biosafety protocol (http://www.bch.biodic.go.jp/english/law.html).

Japan has a number of wild *Rosa* species, such as *Rosa multiflora*, *R. lutea* and *R. rugosa.* These have also been utilized to breed *R. hybrida*. Japanese regulators were therefore concerned that the pollen from transgenic rose could hybridize with populations of wild roses. It was therefore considered desirable to show that the wild roses do not hybridize with cultivated roses under natural conditions. Though this is predictably unlikely because *R. hybrida* is generally tetraploid whilst wild roses are diploids, a genetic marker is available to allow for detection of hybrids in the field. This marker gene in cultivated rose, which regulates the ever-blooming character derived from *R. chinesis* [77] was not found in thousands of seeds of wild roses found growing close to cultivated *R. hybrida* in Japan, showing that wild roses and *R. hybrida* are unlikely to hybridize under natural condition.

WKS82-130-4-1 and WKS82-130-9-1 plants did not show significant difference from their host in terms of growth character, morphology, phytoalexin assays and so on except flower colour in glasshouse and field trials. Using *in situ* hybridization techniques and crossing experiments, it was possible to show that only L1 cells (the epidermal cells where anthocyanins are localized) of the transgenic rose plants contained the transgenes and L2 cells (producing pollen) did not. The lack of the transgene in the pollen cells is obvious barrier to potential gene flow to wild roses. On the basis of these results, general release permissions were issued for the two rose lines in Japan. In late 2009, one of these lines has been commercially produced and launched in Japan as the Suntory blue rose APPLAUSE^™^.

### 4.5. Genetic Modification for Red Flowers

Accumulation of pelargonidin based anthocyanins in flowers confers an intense red or orange colour, which is very desirable commercially. Attempts have therefore been made to obtain such colours by genetic engineering. In petunia (a variety accumulating cyanidin based anthocyanins), down regulation of the F3′H gene in conjunction with over-expression of rose *DFR* yielded transgenic petunia whose flowers accumulated pelargonidin based anthocyanins [78]. Down regulation of F3′5′H and F3′H genes and over-expression of pelargonium DFR in blue torenia (which normally produces delphinidin based anthocyanins) resulted in transgenics with pink flowers accumulating anthocyanins based on pelargonidin (manuscripts in preparation). Down regulation of F3′H and flavonol synthase and overexpression of gerbera DFR in tobacco yielded red coloured flowers accumulating pelargonidin based anthocyanins [79]. However down regulation of F3′H was not complete as the transgenic tobacco still produced significant amount of cyanidin-based pigments. In osteospermum a two step transformation method has been used in which down regulation of F3′5′H was followed by overexpression of gerbera DFR. This particular transformation resulted in pelargonidin accumulation in flowers and a flower colour change toward red. In contrast, overexpression of gerbera or strawberry DFR alone did not yield flowers accumulating pelargonidin [80]. As pelargonidin was accumulated even though F3’H activity was not down regulated, F3′H activity is weak in osteospermum.

Some cut flower species such as gentian and iris do not accumulate pelargonidin derived pigments and thus lack flowers with an intense red colour. Red gentian cultivars (Showtime Diva, Showtime Starlet, Showtime Spotlight) have been made by interspecies hybridization [81]. Genetic modification methods should be an alternative solution to achieving these new flower colours.

Though down-regulation of the F3′H and F3′5′H genes in combination with over expression of a correctly identified DFR gene should generate gentian flowers producing pelargonidin based pigments. Sophisticated optimization of gene expression or pathway engineering may be necessary to accumulate a large amount of pelargonidin, and so an intense red flower colour. Furthermore, *Agrobacterium*-mediated transformation of gentian occurs at a low efficiency and the promoter of the 35S cauliflower mosaic virus, a commonly used constitutive promoter, is prone silencing by methylation in gentian [82]. Such difficulties often occur in non model species but may be overcome. For example, a pale flower coloured gentian, which produced less acylated anthocyanins was made from a blue one by down regulation of F3′5′H and 5,3′AT. In this case, the *rol*C promoter was utilized [83].

## 5. Concluding Remarks

We have described some successful examples of colour modification. Recently genes regulating flower colour such as pH and metal transport genes have been identified and these may prove useful molecular tools for modification of flower colour in the future. Of course, efficient transformation systems have to be developed for target cultivars of interest and transgene expression also needs to be optimized. A considerable amount of try and error is inevitably required to achieve a satisfactory final result.

In our experience, genetic modification of an ornamental plant can be a successful venture, from both a scientific and a commercial perspective. The “Moon” series of colour modified carnations have been sold now for nearly a decade and millions of flowers have entered the traditional growing, distribution and retail chains for cut-flowers. There is no reason to think transgenic rose flowers will not be equally as readily accepted in the marketplace and to date there has been no negative response from consumers to the genetically modified rose. The transgenic varieties have proven to be genetically very stable during mass scale vegetative propagation and there have been no unexpected effects on either the environment or on the health of those handling the flowers. The beneficial health effects of anthocyanins are in fact well recognized; an understanding that is leading to the development of genetically modified, functional foods, with enhanced anthocyanin profiles [84].

What then is the major obstacle to dozens of other genetically modified ornamental products entering the marketplace, given that the technology for trait manipulation is in place, and that many important horticultural crops can be transformed? The answer lies in the barriers that the regulatory systems in many parts of the world place on the freedom to trial and develop GM varieties of ornamentals. As is the case for cut-flowers, ornamental products are an internationally traded commodity and until there is an internationally agreed, equitable process, for regulating genetically modified plant products it will continue to prove very difficult to release ornamental products, due to the costs and expertise required for commercial development. To ease this burden the regulatory requirements for non-food varieties, such as ornamentals, should be reduced.
